# Relationships Between Spiritual Quotient and Marital Satisfaction Level of Men, Women and Couples Referred to Consultancy Centers of Bandar Abbas 

**Published:** 2013

**Authors:** Eghbal Zarei, Tahereh Ahmadisarkhooni

**Affiliations:** 1 Department of Family Consultancy, Hormozgan University,Km.9 Old road of Minab, Hormozgan University, Bandar Abbas, Hormozgan, Iran.

**Keywords:** Consultancy, Couples, Marital Satisfaction, Spiritual Quotient

## Abstract

**Objective:** The aim of this research is to determine the relationship between Spiritual Quotient parameters including understanding, life origin, and spiritual life and marital satisfaction of couples in Bandar Abbas City.

**Methods:** It is descriptive correlational study. 150 couples referred to consultancy centers of Bandar Abbas City were selected by accessible sampling method. We utilized Spiritual Quotient Questionnaire and Marriage Satisfaction Questionnaire (ENRICH) which both have high reliability and validity levels. We calculated men, women and couples’ scores in the questionnaires.

**Results:** According to the findings; among all parameters of Spiritual Quotient, spiritual life had the strongest correlation with spiritual quotient (r=0.282 and r=0.277 for men and women; P<0.01 for both). Meanwhile, there were not any significant relationship between couples’ understanding and origin of life and their marital satisfaction.

** Conclusion:** Overall, we can conclude that training according to cultural conditions as well as promoting couples’ spiritual quotient can be utilized to improve the quality of marital life of couples.–More studies should be conducted for further evaluation of the relationship between SQ and marital satisfaction. The results can be used for helping couples in increasing their marital satisfaction.

**Declaration of interest:** None

## Introduction

Human being has been always interested in conducting researches and investigations about various specifications and types of the concept of intelligence. One aspect of intelligence is specified under the title of “Spiritual Quotient” (SQ) which is the focus of attention of psychologists who study on the field of religion and spirituality. No organized and systematic study has been performed to determine the specifications of different aspects of SQ and their relationship with other variables including marital satisfaction. SQ is beyond the physical relations of individuals with their surrounding environment. It may enter higher dimensions of life enabling individuals to find hidden resources of love and benefits in disorders & high stress daily life (1).

Sometimes marriage faces some difficulties due to the lack of couples’ knowledge about mental abilities, value systems and behavioral relations. When both husband and wife conclude that they lack mutual factors, their marriage connections would be weakened and finally destroyed (2). Gardner (1999) has evaluated different concepts and content of SQ. He explained SQ as an aspect of intelligence responsible for explaining secrets of life and presenting final questions about life mysteries (3). Unlike Intelligence Quotient which can also be considered for computers and Excitement Quotient which is applicable in high level mammals; SQ is only applicable for human beings. Individuals use SQ to improve their personal interests, preserving values and achieving their goals. SQ enables individuals in utilizing their abilities for overcoming difficulties. SQ is the foundation of personal beliefs and has a significant role in individuals’ reactions and forming their lives (4).

SQ is related to individuals’ lives and their relations with life and the environment. SQ is a means of good understanding of deep spiritual questions and having internal attitude about different aspects of intelligence. SQ means good knowledge of spirituality as a field or an innovative force of life. SQ means having complete and internal knowledge and deep information about body, materials, mentality and spirituality. SQ is like mental and personal abilities. It is related to a person, inter-personal relations and also mentality. It is complete knowledge about people themselves and their relationship with other persons and all creatures. SQ would developed by exercise. Different cultures explain it as love, servitude or worship. SQ is related to excitement quotient; therefore as a spiritual exercise it includes inter-personal interactions. A way of increasing individuals’ knowledge about their internal spiritual life is paying more attention to the feelings and kindness. SQ depends on personal characteristics and recognition of the relationships among concepts, belief and behavior. Most people are responsible for their behaviors but not against their doubts and concepts. Increasing individuals’ SQ needs more education and training. When people seek for meaning of different questions like who am I? Why am I here? etc., we are trusting SQ (5).

Amram (2005) believes that SQ includes meaning and duties in life, holy feeling of life, balanced understanding of materials and beliefs about the better world (6). 

Mc Mullen (2003) believes that some values including brevity, integration, witness and kindness are included in SQ. He also believes that there is a relationship between insight and SQ and in contrast, stress is against intuition. He introduces calm attention as one of the ways for increasing insight. Moole also believes that stress is an increasing and end-less effort against any delays in decision making (7).

In a research conducted by Yaghoubi et al (2007) it was concluded that there is a significant relationship between SQ and mental health. There were not significant differences between SQ of men and women but they are different regarding mental health (8). Another research conducted by Ghobari Bonab et al (2007) revealed that SQ includes a type of compatibility and solving-problem behavior. As a result it includes the highest levels of development of different recognizing, behavioral, excitement and inter-personal scopes which may enable the individuals in coordination with surrounding environment and finding an internal/external integration (1). 

Abedi and Sorkhi (2009) concluded that there is a significant inverse relationship between SQ and depressed mind and also a significant direct relationship between SQ and extrovert personality factors and also being contentious. There is not a significant relationship between SQ and reflection and being acceptable (9). 

Emmons (2000) studied on effects of SQ on personal health and observed that spirituality is associated with lower diseases prevalence and longer life. In addition, spiritual people would response to treatments better and go with damages easier. Depression rate is lower among these people (5).

This research was conducted for further evaluation of the relationship between SQ and marital satisfaction of couples who had referred to consultancy centers of Bandar Abbas City. For this purpose, we tried to specify the relationship between sub-criteria of SQ including spiritual life and understanding and their relationship with life origin and marital satisfaction. Regarding previous researches, we can consider SQ as an important factor for increasing couples’ marital satisfaction level. Thus we conducted this study to determine the relationship between SQ and marital satisfaction. Results of this study may help couples in endeavor for increasing their satisfaction of life and then family life.

## Materials and methods

This is a descriptive correlation study. Researcher did not perform any interventions and only tried to explain any relationship between SQ and marital satisfaction.

***Population, sample and sampling***

Statistical society of this research comprised of couples referred to consultancy centers of Bandar Abbas City including consultancy center of Family Judicial Complex in Enghelab General Justice Office, Private Consultancy Centers, and Center of interfere in family crisis and Divorce Reduction Center. Couples who were married for at least for 2 years and have one or more children were enrolled in the study. Eventually we selected 150 couples through sampling method. 

***Research tools***

SQ and ENRICH questionnaires were applied for data gathering.

*Enrich marital satisfaction questionnaire *

Olson (1989) developed this questionnaire which comprised of 47 questions and 12 criteria for evaluation of marital satisfaction (10). Mirkheshti (1996) translated the questions to Persian language. After consulting with experts, he omitted the questions which were in contrast with Iranian culture. Eventually he formed a questionnaire with 47questions. Then he studied the questionnaire in a trial on 30 individuals. Some questions were incomprehensible to the participants and were modified. In the end, the reliability of the Persian questionnaire was approved by the experts. Correlation coefficient of “Enrich questionnaire” with familial satisfaction reported to be about 0.41 to 0.60 and with life satisfaction criteria to be about 0.32 to 0.41 which demonstrate its structural validity. Enrich questionnaire may detach satisfied and-dissatisfied couples which is the rationale for validity of our study. Furthermore, Mirkheshti (1996) has studied on 60 persons and calculated the Cronbach alpha coefficient to be 0.92 which is a high coefficient (11).

*Spiritual quotient Questionnaire*

Abdollahzadeh along with Keshmiri and Arab Ahmadi developed the spiritual quotient questionnaire in 2009 and accordingly, the final questionnaire was prepared with 29 questions. To evaluate the validity of the questionnaire, we used factor analysis rather than superficial content validity upon the comments of experts. The correlation between questions was more than 0.3. Regarding reliability of the test, alpha in primary step was 0.87 and 0.89 in final step of reliability. For estimating validity of the questionnaire we used both content and simultaneous validity as well. Correlation coefficients of both parameters (any relationship with life origin and spiritual life) were 0.416 and 0.583 in females and 0.638 and 0.387 in males respectively. This shows the appropriate time validity of the spiritual quotient questionnaire. Reliability of this research was evaluated and using Cronbach alpha. Cronbach alpha for the entire questionnaire, sub-criteria of spiritual life, understanding and any relationship with life origin were 0.94, 0.91 and 0.89 respectively. This shows the suitable reliability of the questionnaire (12).

## Results

Results of this research are presented in two parts. Firstly, table 1 depicts descriptive results including mean and standard deviation of couples’ scores of marital satisfaction and SQ. Furthermore table 2 depicts the results of statistical tests including Pearson correlation coefficients.

Pearson simple correlation coefficient is used for evaluation of relationship between marital satisfaction level and couples’ spiritual quotient parameters. The data depicted in table 2shows that there is a significant direct relationship between spiritual life parameters and marital satisfaction (r=0.282; p<0.01 and n=150). There is also a direct relationship between understanding parameter and life origin and marital satisfaction but it wasn’t statistically significant.

**Table 1:** Mean and standard deviation of scores of couples, females and males

**Men**		**Women**		**Couples**		
**Standard** **deviation**	**Mean**	**Standard deviation**	**Mean**	**Standard ** **deviation**	**Mean**	**Criteria index**
17.75	119.95	15.75	112.15	33.50	233.1	**Couples’ marital satisfaction**
12.82	113.47	12.50	113.3	25.32	226.77	**Couples’ spiritual quotient **
4.98	52.47	5.03	52.73	10.01	105.2	**Understanding & relation with life origin of couples**
9.94	61	7.94	60.58	17.88	121.58	**Spiritual life of couples**

**Table 2:** Relationship between spiritual quotient & couples’ marital satisfaction

		**Understanding and any relation with life origin**	**Spiritual life**
**Couples’ marital satisfaction**	Pearson correlation coefficient	0.123	** 0.282
	Significance level	0.133	0.01
	Qty	150	150

* p<0.05

** p<0.01

According to the results in table 3, there was a relationship between understanding parameters, life origin, spiritual quotient of women and their marital satisfaction (r=0.025 and 0.122 respectively) and also a direct relationship between two aforementioned parameters and marital satisfaction but was not statistically significant.

**Table 3:** The relationship between spiritual quotient and marital satisfaction in women

		**Understanding & any relation with life origin**	**Spiritual life**
**Women’s marital satisfaction**	Pearson correlation coefficient	0.025	0.122
	Significance level	**0.765	*0.136
	Qty	150	150

* p<0.05

** p<0.01

Results of table 4 shows that there is a significant direct relationship between spiritual quotient in men and their marital (r=0.277; p<0.01 and n=150). The results also show that there is a direct relationship between understanding parameters and life origin, and men’s marital satisfaction level (r=0.143) but was not statistically significant. Although the relationship was not statistically significant, but it doesn’t mean that such variable could not estimate the marital satisfaction. Generally, the relationship between SQ and marital satisfaction is more significant in men, which means that the men with higher SQ have higher marital satisfaction level.

**Table 4:** The relationship between spiritual quotient and marital satisfaction in men

		**Understanding & any relation with life origin**	**Spiritual life**
**Men’s marriage satisfaction**	Pearson correlation coefficient	*0.143	** 0.277
	Significance level	0.081	0.01
	Qty	150	150

* p<0.05

** p<0.01

## Discussion

The aims of previous studies were recognizing the effects of SQ on marital satisfaction and their relationship. With the same goal, the present study was conducted and showed important results regarding this field. According to the findings of this study, there is a significant relationship between marital satisfaction and couples’ SQ. Couples required SQ in their marital relationships to achieve marital satisfaction. The strongest correlation was between men and women’s spiritual life and their marital satisfaction. Although there is not any direct relationship between results of preceding researches and the current study, but since both SQ and marital satisfaction are specified in preceding studies, one can find them analogous and notice some similarities between them. Results of our study were comparable with findings of Mahanian Khameneh, Borjali and Salimi Zadeh’s study (2006) as they reported a significant correlation between excitement quotient and marital satisfaction. Therefore it seems that various parameters including excitement quotient and excitement self-conscious, social and self-skills have significant effects on couples’ marital satisfaction. Excitement quotient and marital satisfaction have also significant effects on individuals’ job satisfaction (13).Furthermore; results of Khodayari Fard, Shahabi and Akbari Zard Khaneh’s study (2007) demonstrated that there is a significant direct relationship between individuals’ religious attitudes and their marital satisfaction. They also reported that among the four sub-criteria, marital compatibility and religious attitude had the strongest relationship with couple’s satisfaction (14). Results of our study were also comparable with findings ofLotfi and Sayyar (2008) as they reported that there is a significant relationship between couples’ SQ and mental health (15). Moreover, Booj Mehrani (2008) showed that there is a relationship between individuals’ SQ and alcohol addiction, which is comparable with results of our study (16). As demonstrated in a research performed by Abedian Sorkhi (2009), there is a significant direct relationship between individuals’ SQ and extrovert personal factors and conscience (9). According to considerations of Mack Howk (2002) as reported by Nasel (2004), compared with non-religious education and realistic knowledge, SQ has a close relationship with individuals’ attitude and satisfaction. SQ may develop all concepts individuals and assists in making deep relations which would enrich people’s relations. Furthermore, spiritual growth and moving toward blossoming are mostly related to individuals’ SQ (17). Yung’s research (2006) showed that age, beliefs, high records and childhood spirituality are fundamental and effective factors on individuals’ SQ which is consistent with present study (18). Also as reported in Zargar et al. study (2008), individuals with lower levels of religious attitudes are more prone to addiction (19). Results of Sullivan’s study (2001) showed that persons with higher religious attitude have higher marital satisfaction compared to individuals with lower religious attitude. This is in tandem with results of our study, because both SQ and marital were considered in the study (20). Hanler and Genchoz (2005) stated that there is significant relationship between religion and familial conditions. Thus having religious beliefs may increase couples’ marital satisfaction and do not increase couples’ marital problems. Those findings are consistent with results of current study (21). Orathinkal and Vansteewegen (2006) compared couples who had married one time with the ones who had married more than one time and concluded that religious beliefs have positive effects on couples’ marital satisfaction (22) .According to the studies of Shiota and Levenson (2007), any similarities between couples in different dimensions including social-economic situation, academic and educational conditions, age, nationality, religion, body attractions well as intelligence may increase couples marital satisfaction which is in compliance with findings of our research (23).Flowers’s study (1998) showed that religion is more important for men and having equal rights is more important for women. These two aforementioned factors have significant effects on marital satisfaction, which is concordant with results of our study (24).

Mc Mullen (2003) believes that SQ is related to asking more than replying. This means that only those with a lot of questions about themselves and life and surrounding world could make desired relationship with their environment and thus with surrounding persons (7).Therefore one can conclude that similar to other aspects of intelligence, SQ may also upgrade personal functions of individuals. Consequently, based on results of previous and our studies, there is a significant relationship between peoples’ SQ and their marital satisfaction. Generally we found a significant direct relationship between SQ and marital satisfaction in couples, women and men. Although there are few domestic and foreign researches in this field; and out study was almost a novel one, but such a relationship was anticipated with regard to theoretical basics of spirituality and SQ.

The relationship between individuals’ SQ and their marital satisfaction is complex and its details have not completely been understood yet. Therefore it is important to conduct studies for better understanding of this complex relationship. Eventually we may conclude that for improving marital life quality of couples, men and women we can upgrade training of SQ aspects with regard to cultural and religious conditions of our society with more attention to sub-criteria of spiritual life and. Performing further trial researches to evaluate the effects of individuals’ SQ on increasing couples’ marital satisfactions necessary to help couples to have higher marital satisfaction and life quality and to live with calmness and confidence.

## Authors, Contributions

 ZE conceived and designed the study, helped in interpretation and statistical analysis of the data, and contributed in drafting the manuscript. AST participated in collecting the data and drafting the manuscript, took part in conceiving and designing the study and also helped in interpretation of the results. Both authors read and approved the final manuscript.
